# Supplementary material for: Aberrant DNA Methylation, Expression, and Occurrence of Transcript Variants of the ABC Transporter ABCA7 in Breast Cancer
Source: Cells. 2023 May 24;12(11):1462. doi: 10.3390/cells12111462 (PMC10252461; doi:10.3390/cells12111462)
Supplement: Supplementary file 1 [file cells-12-01462-s001.zip › cells-2283225-supplementary.pdf]

cells

Supplementary Figures

Aberrant DNA Methylation, Expression, and Occurrence of Transcript Variants of the ABC transporter *ABCA7* in Breast Cancer

Katja Zappe [1], Antonio Kopic [1], Alexandra Scheichel [1], Ann-Katrin Schier [1], Lukas Emanuel Schmidt [1], Yasmin Borutzki [1,2], Heidi Miedl [3], Martin Schreiber [3], Theresa Mendrina [2,4], Christine Pirker [4], Georg Pfeiler [5], Stefan Hacker [6], Werner Haslik [6], Dietmar Pils [7], Andrea Bileck [1,8], Christopher Gerner [1,8], Samuel Meier-Menches [1,2,8], Petra Heffeter [4], Margit Cichna-Markl [1,*]

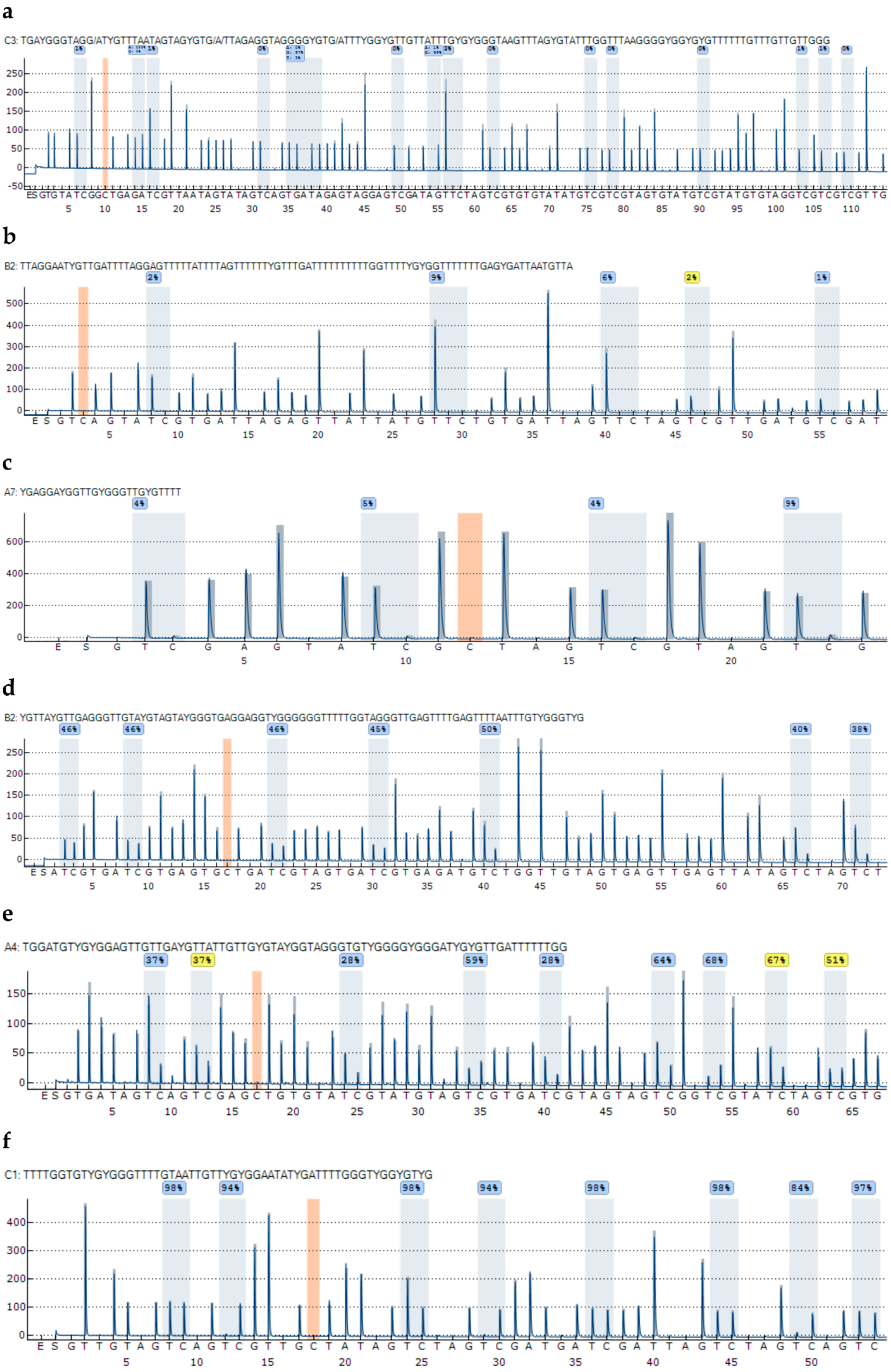

Figure S1. Representative pyrograms obtained for the *ABCA7* regions for (**a**–**d**) tumor tissue from patient 14 and (**e**–**f**) MDA-MB-231 cell line. Assays: (**a**) prom, (**b**) A, (**c**) B, (**d**) C, (**e**) D, and (**f**) E. Peaks highlighted in blue-grey indicate the position to analyze, grey bars represent the expected dispensation heights according to the dispensation order (histogram). Methylation levels of the CpG dinucleotides is shown above the respective

position to analyze, for region prom, nucleotide frequencies for rs3764642 (NC_000019.10:g.1040047G>A, MAF: 0.448(A); G>C, MAF: 0.000(C) manual discrimination), rs531508435 (NC_000019.10:g.1040065G>A MAF:<0.001(A); G>T MAF: 0.000(T)), and rs549725064 (NC_000019.10:g.1040081G>A MAF:< 0.001(A)) are additionally shown. Blue and yellow colors indicate the quality of the result (blue passed, yellow check). The position highlighted in orange allows for assessment of the efficiency of bisulfite conversion.

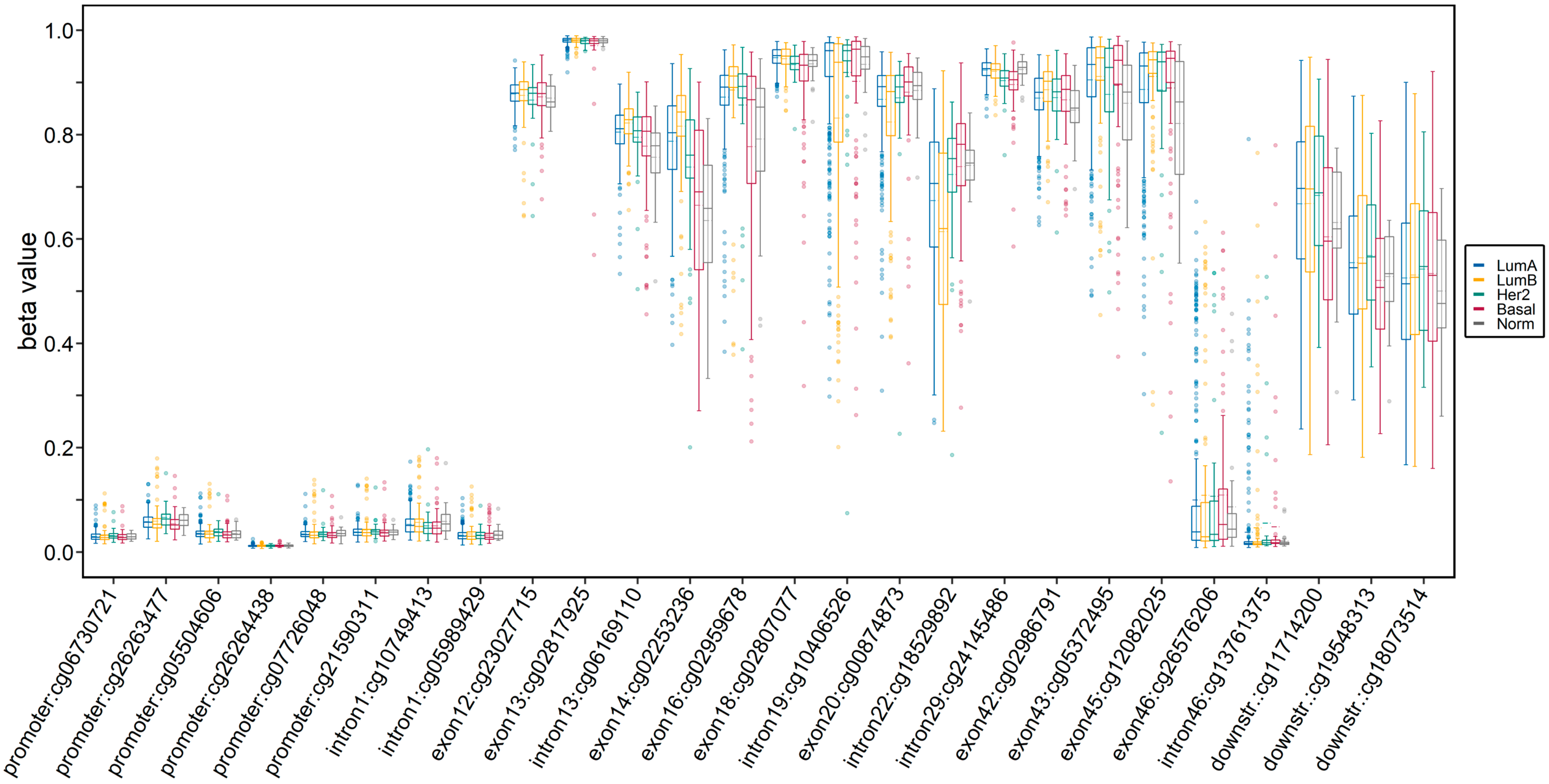

Figure S2. Beta values for individual CpGs covered with the Infinium Human Methylation 450K BeadChip in the promoter, in *ABCA7* gene regions, and directly downstream (downstr.) of exon 47 (< 200 bp) for 683 TCGA breast cancer tissues samples (372 luminal A, 125 luminal B, 44 Her2-positive, 112 basal-like, and 30 normal-like molecular subtypes).

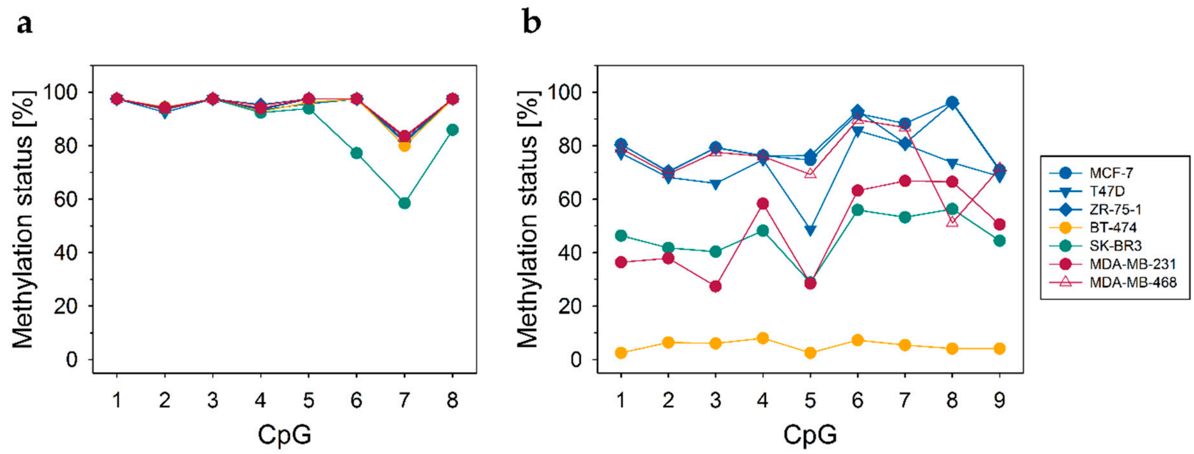

Figure S3. DNA methylation status of (**a**) CpGs 1–8 (CpG 7 = cg23027715) in exon 12 and (**b**) CpGs 1–9 at the exon 6/intron 6 boundary for seven breast cancer cell lines of luminal A (blue), luminal B (yellow), Her2-positive (green), and triple-negative (red) molecular subtype. Each data point shows the arithmetic mean of at least two technical replicates.

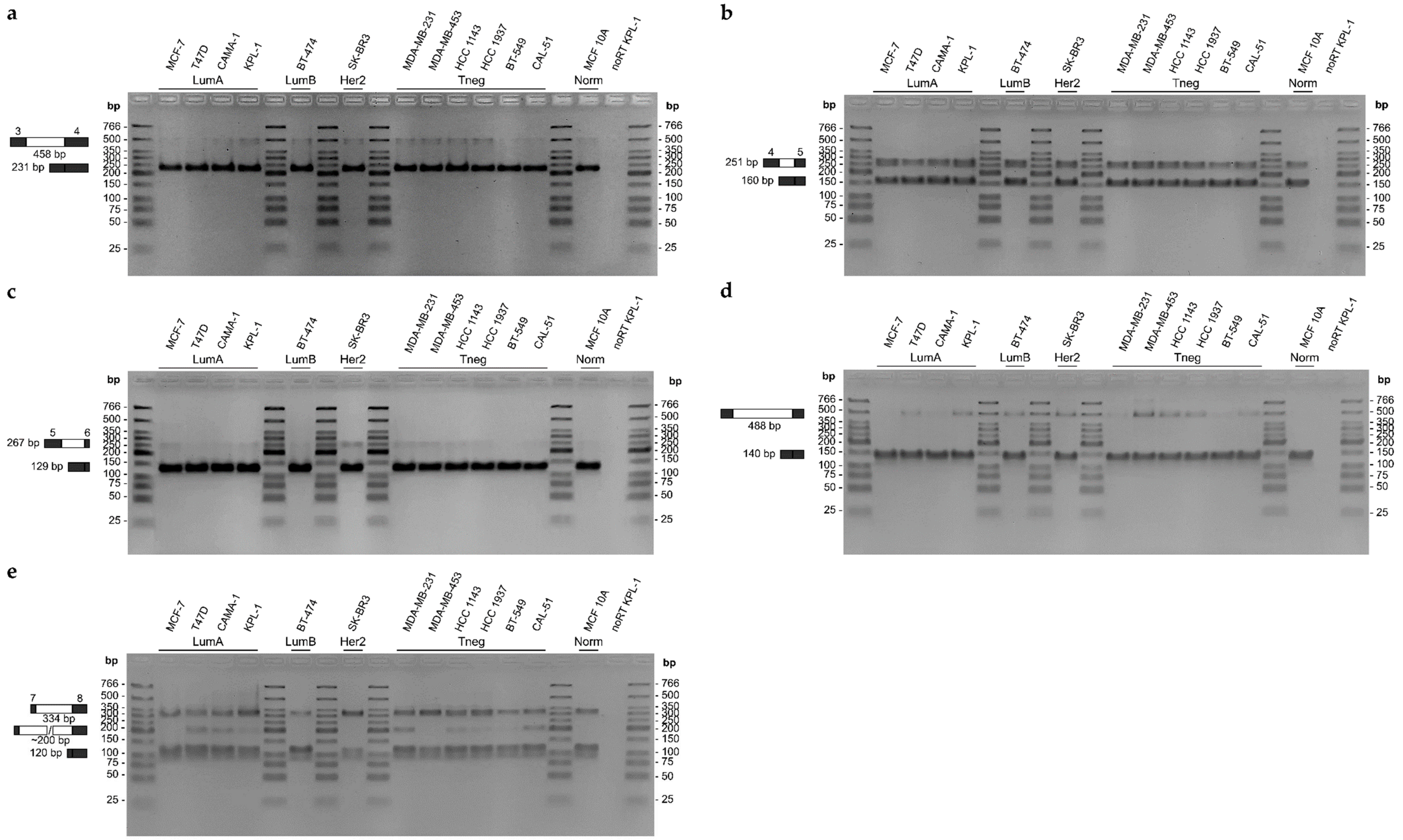

Figure S4. Agarose gel electrophoresis analysis of PCR products obtained by using intron-flanking primer sets: (**a**) exon 3–(intron 3)–exon 4, (**b**) exon 4–(intron 4)–exon 5, (**c**) exon 5–(intron 5)–exon 6, (**d**) exon 6–(intron 6)–exon 7, and (**e**) exon 7–(intron 7)–exon 8. Considering splicing donor GT and acceptor AG sites, alternative termination of intron 7 could lead

to PCR products of 197 bp (ending with TAG), 206 bp, or 222 bp, alternative intron 7 start in PCR products of 203 bp or 207 bp in length. Schemes show exons (dark gray fill), introns (white fill), and the expected PCR product length.

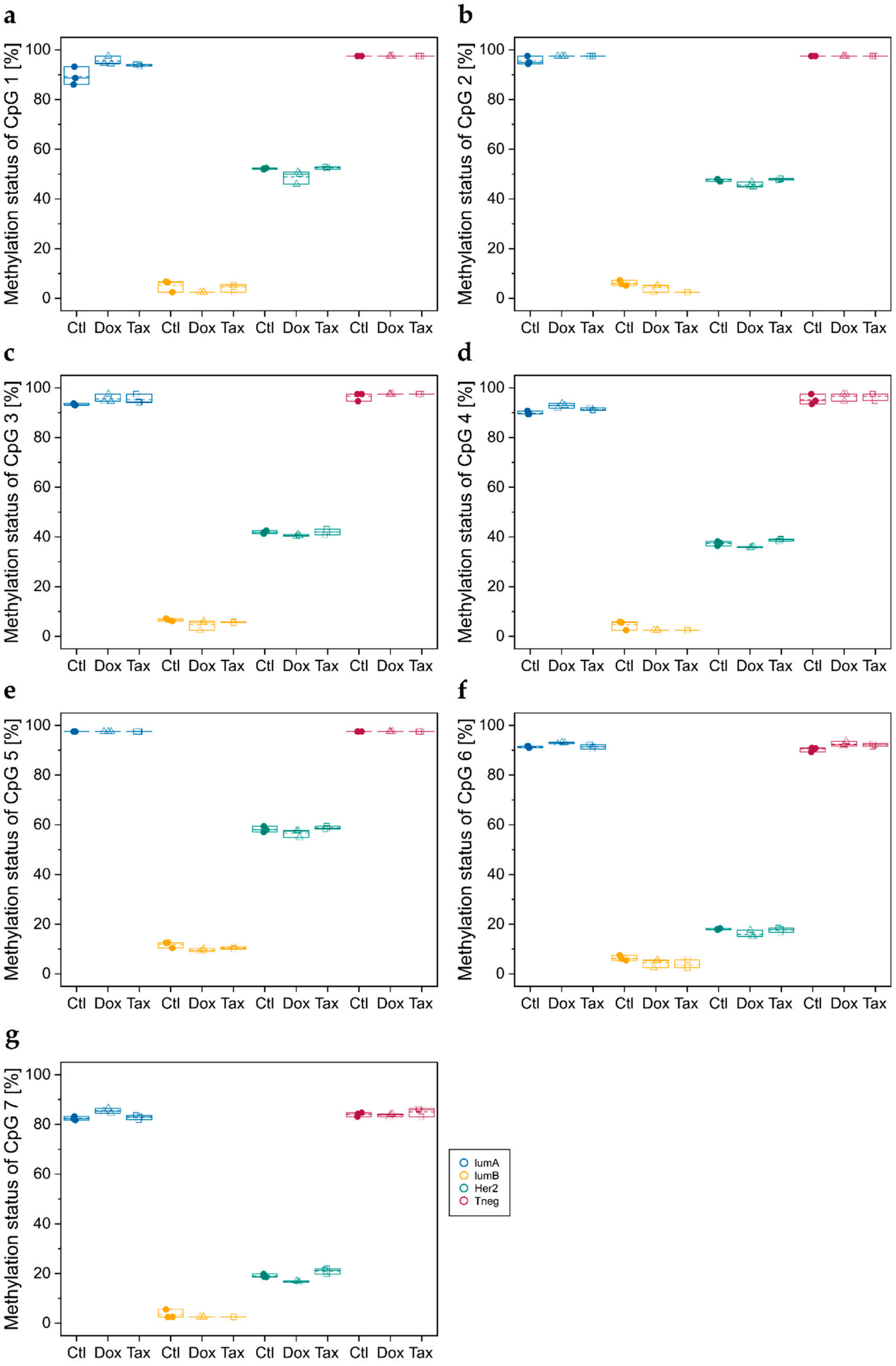

Figure S5. Distribution of the DNA methylation status of CpGs 1–7 at the *ABCA7* exon 5/intron 5 boundary for treated cell lines. (**a**) CpG 1, (**b**) CpG 2, (**c**) CpG 3, (**d**) CpG 4, (**e**) CpG 5, (**f**) CpG 6, and (**g**) CpG 7. Ctl: untreated, Dox: doxorubicin, Tax: paclitaxel, molecular subtypes LumA: luminal A (MCF-7); LumB: luminal B (BT-474); Her2: Her2-positive (SK-BR3); Tneg: triple-negative (MDA-MB-231). Each data point shows the arithmetic mean of at least two technical replicates. Solid line: median, dashed line: arithmetic mean. No significant difference was found between treated and untreated cells.
